# Probing Downstream Olive Biophenol Secoiridoids

**DOI:** 10.3390/ijms19102892

**Published:** 2018-09-23

**Authors:** Ganapathy Sivakumar, Nicola A. Uccella, Luigi Gentile

**Affiliations:** 1 Department of Engineering Technology, College of Technology, University of Houston, Houston, TX 77204, USA; 2 IRESMO Foundation Group, via Petrozza 16A, 87040 Montalto Uffugo, Italy; nicola.uccella@unical.it; 3 Department of Mechanical, Energy and Management Engineering (DIMEG), University of Calabria, P. Bucci 42C, 87036 Rende, Italy; 4 Chemistry and Chemical Technology Department, University of Calabria, P. Bucci 12C, 87036 Rende, Italy; luigi.gentile@biol.lu.se; 5 Molecular Ecology, Microbial Ecology and Evolutionary Genetics (MEMEG) unit, Department of Biology, Lund University, 22362 Lund, Sweden

**Keywords:** antioxidant, biophenols, olive oil, oleuropein, tyrosol, hydroxytyrosol, TNDO

## Abstract

Numerous bioactive biophenol secoiridoids (BPsecos) are found in the fruit, leaves, and oil of olives. These BPsecos play important roles in both the taste of food and human health. The main BPseco bioactive from green olive fruits, leaves, and table olives is oleuropein, while olive oil is rich in oleuropein downstream pathway molecules. The aim of this study was to probe olive BPseco downstream molecular pathways that are alike in biological and olive processing systems at different pHs and reaction times. The downstream molecular pathway were analyzed by high performance liquid chromatography coupled with electrospray ionization mass spectrometry (HPLC-ESI/MS) and typed neglected of different overlap (TNDO) computational methods. Our study showed oleuropein highest occupied molecular orbital (HOMO) and HOMO-1 triggered the free radical processes, while HOMO-2 and lowest unoccupied molecular orbital (LUMO) were polar reactions of glucoside and ester groups. Olive BPsecos were found to be stable under acid and base catalylic experiments. Oleuropein aglycone opened to diales and rearranged to hydroxytyrosil-elenolate under strong reaction conditions. The results suggest that competition among olive BPseco HOMOs could induce glucoside hydrolysis during olive milling due to native olive β-glucosidases. The underlined olive BPsecos downstream molecular mechanism herein could provide new insights into the olive milling process to improve BPseco bioactives in olive oil and table olives, which would enhance both the functional food and the nutraceuticals that are produced from olives.

## 1. Introduction

Olive oil and table olives are a major part of the traditional food in the Mediterranean Aliment Culture (MAC) [1]. Olives and olive oil have been found to naturally protect against stroke, degenerative and cardiovascular diseases as well as cancer and obesity [2,3,4,5,6,7,8,9]. Extra virgin olive oil is one of the essential sources of monoenoic fatty acids and biophenol (BP)-based bioactives [10,11]. The major BPs from olive products include phenolic acids and alcohols as well as secoiridoids, while the minor contents are flavonoids and lignans [12]. The primary olive fruit biophenol secoiridoids (BPsecos) are oleuropein, ligstroside, and their demethylhomologues, which may have potential anticancer effects [13,14]. Olive leaves contain a higher concentration of BPs with secos of 1450 mg/100 FW (fresh weight) compared to the olive fruit and oil, which have 110 mg/100 g and 23 mg/100 mL, respectively [9]. Oleuropein and ligstroside aglycones, oleacein, oleocanthal, hydroxytyrosil-elenolate, tyrosil-elenolate, oleoside-11-methyl ester, elenoic acid, hydroxytyrosol, and tyrosol are downstream pathway bioactives from oleuropein and ligstroside precursors [1,2,3,4,5,6,7,8,9,10,11,12,13,14,15]. Oleuropein is the principal BPseco in green olive fruits, leaves, and table olives, whereas olive oil is rich in its downstream metabolites [16,17,18]. Oleuropein causes most of bioactive functions and contributes to the food taste and the prevention of several lethal human diseases [19,20,21,22,23,24,25,26]. Moreover, oleacein and oleocanthal are downstream forms of decarbomethoxy-oleuropein and decarbomethoxy-ligstroside, respectively, via aglycones to dialdehydes formation, which are responsible for the intensity of the hedonic-sensorial response to pungency and bitterness in olive oil and table olives [27,28,29]. Similarly, hydroxytyrosol and tyrosol are downstream bioactive metabolites of oleuropein and ligstroside, respectively [16]. These olive bioactives exert antioxidants via free radical processes and electrophilic molecular dynamics. RNA sequencing and MS analysis has previously revealed that oleuropein biosynthesis is involved in the metabolic combination of mevalonic acid pathway for oleoside and phenypropanoid for HO-aromatics [16,17,30].

**Figure 1:**
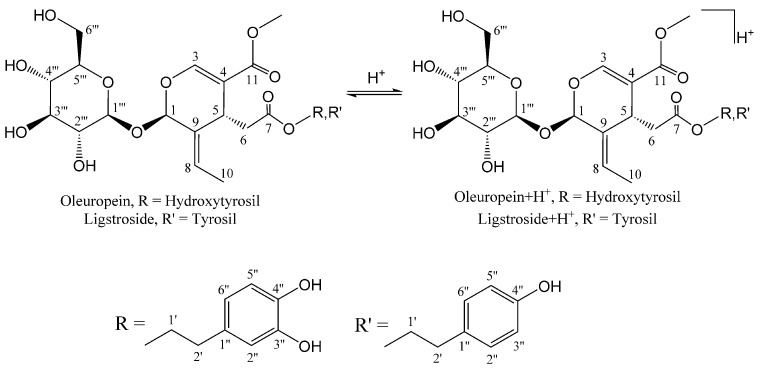
Molecular pre-equilibrium of olive biophenol secoiridoids (BPsecos) and H_3_O^+^.

Olive and human gut microbiota β-glucosidases play critical roles in BPseco downstream conversion during olive fruit maturation, olive mill processing, and the biological metabolism that is responsible for the potential bioactives of extra virgin olive oil and table olives [31,32]. Additionally, olive BPseco pathways to the cleavage of bis-acetal bonds within glucose and secoiridoid subunits and promotes acid methanolysis [33]. Although molecular dynamics and Austin Model 1 (AM1) level computational mapping of oleuropein have been developed, the downstream mechanisms are not fully understood [16,17]. This is one of the drawbacks of important bioavailable olive BPsecos, their concentration present in olive products, and their biomolecular structures [34]. Acid-catalyzed oleuropein downstream pathways, however, could evolve via dynamic pre-equilibrium to oleuropein + H^+^ isomers (Figure 1). The objective of this study was to probe olive BPsecos downstream pathways that are involved alike in human digestion and in processing of olive oil and table olives at different pHs and reaction times using HPLC-ESI/MS and typed neglected of different overlap (TNDO) computational calculations. The results we obtained could provide a new tool for probing olive BPseco-made bioactives and their antioxidant, chemo-preventer, and xenohormetin existence in olive oil, table olives, and olive leaves. This could result in improved guidelines for more effective olive milling and table olive processes that would increase the functionality of olive-based foods and nutraceuticals.

## 2. Results and Discussion

### 2.1. HPLC-MS Analysis

Olive *Cassanese* fruits yield an average of 3.25 g/kg BPseco mixtures during ripening cycles [35]. This cultivar is very common in the MAC eating pattern and is commercially essential for extra virgin olive oil and table olives produced in the region. This cultivar is an important source of olive BPs, which encompasses several BPsecos and has a bitter, astringent flavor with pungency [36,37,38,39]. The level of bitterness in olive oil from various cultivars is a result of BPseco downstream products [40,41,42,43]. *Cassanese* cultivar fruits have a high concentration of oleuropein that measures 10 mg/g dry weight during August to September; however, this decreases as the fruit ripens [15]. Several techniques have been reported to improve olive oil shelf life, flavor, and aroma [44]. Additionally, the metabolism and transcriptional profiles of this cultivar fruit BP have been characterized [45]. In *Cassanese* fruit extracts, the most abundant HPLC peak correspond to oleuropein, followed by oleuroside and isomer; this result has been confirmed by ESI/MS [15].

**Figure 2:**
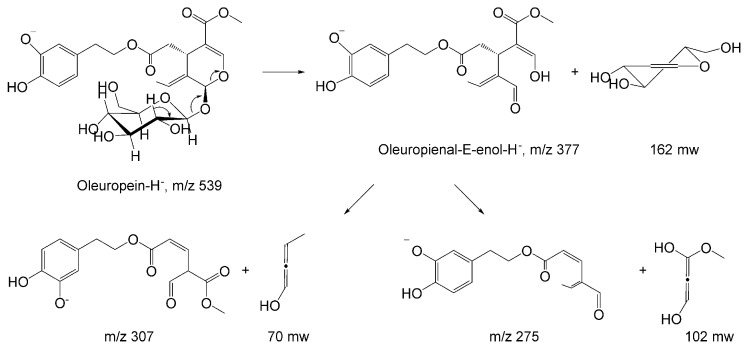
Collision activated dissociations (CAD) unimolecular reactions of oleuropein pseudo-molecular ions, *m*/*z* 539.

Pseudomolecular anions corresponding to *m*/*z* 539 display unique peaks of ν^−^ ESI-MS spectra. ν^−^ MS/MS analysis of oleuropein revealed pK_1_a 9.25 and pK_2_a 13.00 at hydroxytyrosil moiety, *m*/*z* 539 anions undergoing 162 mu loss, and glucose-H_2_O to *m*/*z* 377 as oleuropeinal-E-enol anion (Figure 2). An identical pattern was recognized for oleuroside–H^−^ in ESI-MS/MS, dissociating to *m*/*z* 377, *m*/*z* 307, and *m*/*z* 275, with *m*/*z* 223 and *m*/*z* 179 [16].

### 2.2. H_3_O^+^ and OH^−^ Simulation and Characterization

**Figure 3:**
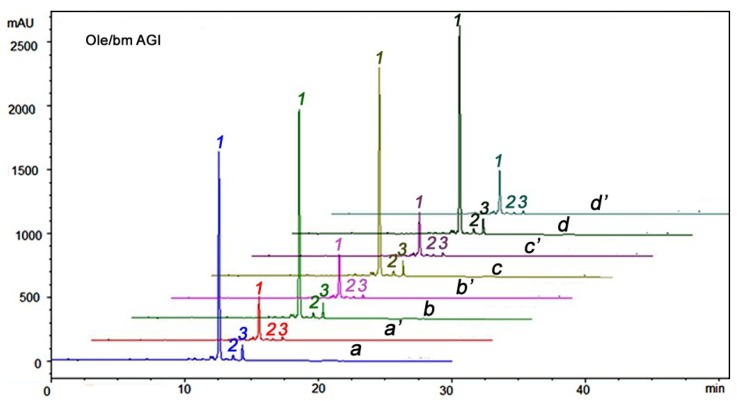
Olive BPseco HPLC/UV profiles; initial stage: a, λ = 240 nm and a′, λ = 280 nm; b and b′: pH = 4.2; t = 120 min; T = 25 °C; c and c′: pH = 1.5; t = 240 min; T = 37 °C; d and d′: pH = 6.0 and 8.0; t = 120 and 120 min; T = 37 °C, under experimental simulations of human digestive and olive processing conditions. Peak 1: oleuropein; peak 2: isomer unidentified; peak 3: oleuroside.

Olive milling simulation showed that olive BPseco mixtures at pH = 4.2, 120 min, 25 °C remained intact without any hydrolytic reaction (Figure 3). Simulations of human digestive conditions gave identical results. Olive BPseco mixtures were unreactive even at pH = 8.0, 120 min, 37 °C due to experimental micro-aerobic conditions. The resembling conditions were chosen to prevent hydroxytyrosol oxidative degradation to the corresponding *O*-quinone analogue followed by polymerization [16]. This result shows that olive BPseco molecular dynamics must be ascribed to lactic bacteria fermentation during table olive processing [46,47]. The two esters and bis-acetals did not hydrolyze, even under olive oil processing conditions. Iridoids glucoside analogs quickly hydrolyzed in about one hour in 2 M HCl [48]. Oleuropein reacted within 8 h, refluxing in aqueous CH_3_CN at 80 °C and Er(OTf)_3_ as the Lewis catalyst [49]. Apparent first-order kinetics was observed for other olive BPseco metabolites in iso-osmotic medium containing NaHCO_3_. Olive BPseco C7 esters cleaved after 350 days in darkness and lipid medium at 35 °C under simulated storage conditions and were affected by oil acidity and filtration [50]. Sample aeration produced total hydrolysis and degradation in 40 min or less [51]. Overall, olive BPseco acid-catalyzed and base-catalyzed molecular dynamics showed comparable structural effects. However, the downstream bioactives, such as hydroxytyrosol and olesoside-11-methylester, were only released at pH = 12.7 for 40 min at 25 °C. Additionally, olive BPseco downstream hydrolysis steps were shown to be controlled by Bronsted/Lewis acid–base or enzymatic catalysis [16]. H^+^ transfers as Bronsted acid or unshared pair/empty orbital interactions were alike in the Lewis base that occurred in olive BPsecos downstream pathways. In fact, olive BPseco hydrolytic downstream mechanisms were inferred under the frontier molecular orbital (FMO) mechanism [17].

### 2.3. Typed Neglected of Different Overlap (TNDO) Mapping

**Figure 4:**
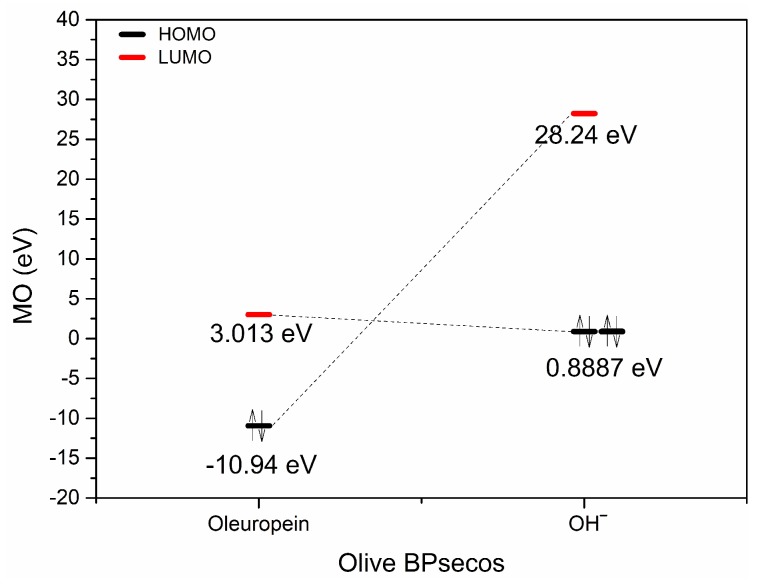
Frontier molecular orbital (FMO) interactions of oleuropein lowest unoccupied molecular orbital (LUMO) and OH^−^ highest occupied molecular orbital (HOMO).

Oleuropein Lewis acid and basic behavior were evaluated by TNDO calculation. These gave new semiempirical merges of molecular mechanics and semiempirical quantum mechanics for olive BPsecos molecular dynamics. TNDO combines olive BPseco atom typing with a basic quantum mechanical method that shows the difference between atoms with different hybridizations that are suitable for secoiridoid-ring conjugated systems. This rapid semiempirical mapping offers more reliable olive BPseco hydrolytic downstream than AM1 [16,17]. Olive BPseco FMOs occurred at the outermost boundaries of the olive bioactive electrons and were closest in energy measurement to molecular reactants, which allowed them to strongly interact. These were the principal factors for determining the occurrence and nonoccurrence of free radical and polar reactions as well as the selective path in intramolecular and intermolecular processes. Oleuropein undergoes competing free radical and polar Lewis type molecular dynamics. Free radical processes occur via HOMO and HOMO-1 interactions. HOMO-2 controls polar reactions like H_3_O^+^ and enzyme catalysis. Olive BPseco LUMOs involve the conjugated seco-ring system on the secoiridoid, similar to Lewis acids, and are energetically the easiest to increase via an increase in electrons through reduction pathways. Polar processes depend on LUMOs for base-catalyzed hydrolysis, which relates to the polar interaction with OH^−^ HOMOs that generate the most probable olive BPseco hydrolytes [16,17]. Figure 4 shows oleuropein/OH^−^ interacting with FMOs, similar to Bronsted behavior. The downstream molecular hydrolytic dynamics of olive BPsecos have been directed at ester functionalities by straight interactions of filled OH^−^ HOMO and LUMO antibonding FMOs at C7=O in competition with C11=O empty orbitals [16]. 

**Figure 5:**
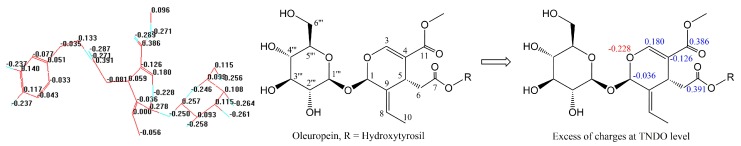
Oleuropein excess of charge on C7, *δ* = 0.391; C11, *δ* = 0.386; C4, *δ* = −0.126; C3, *δ* = 0.180; O2, *δ* = −0.212; C1, *δ* = −0.036.

**Figure 6:**
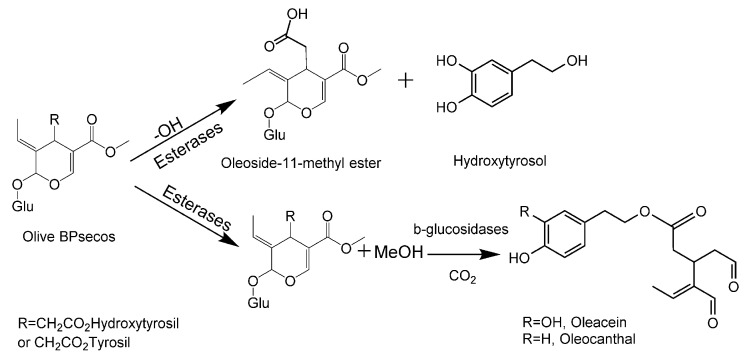
Olive BPseco molecular dynamic sequence under basic and enzymatic catalysis.

Oleuropein empty LUMO at C11=O revealed a possible appropriate reaction site [51]. C11=O carbonyl ester may provide a Lewis acid–base reaction with OH^−^ through the polar interaction of OH^−^ FMOs that filled the electron pair as a Lewis base and revealed the first empty FMO on oleuropein. TNDO calculations exposed oleuropein HOMO = −10.94 eV and HOMO + 1 = −11.75 over hydroxytyrosil moiety, while HOMO-2 = −12.13 eV overlapped the conjugated system –O2–C3=C4–C11=O<―> –O2^+^=C3–C4=C11–O^−^ (Table 1). This was a good electron donor for C11=O carbonyl moiety and explained the Lewis basic character of oleuropein, which destabilizes upon adding H^+^ bonds via bonding disturbance. Large site selectivity guides molecular conversion processes in simulation testing of olive BPseco hydrolytic downstream, which is similar to olive enzymatic degradation at the olive maturity stage [51]. Apparent sterically bulky C7, *δ* = 0.391 ester prevailed on C11, *δ* = 0.386 that less hindered methyl ester, which extensively conjugated to acetal–O2–, *δ* = −0.228 (Figure 5). Electron density affects seco-functionalities, which obscures steric hindrance of olive BPseco groups that induce site selectivity in the basic environment [16]. The overall molecular dynamics of esters at C7=O and C11=O carbonyls in downstream olive bioactive was due to fewer basic and enzymatic catalysis, leading to oleacein and oleocanthal from oleuropein and ligstroside, respectively (Figure 6).

Olive BPseco acid-catalyzed reactions undergo most probable transformations due to downstream hemiacetal on C1 and due to oleuropeindiale. The development of a cascade conversion prevents the process from recovering intermediate products, excluding hydroxytyrosilelenolate. The bioactive intermediates were reactive species of olive BPseco open ring. They rapidly underwent ring closure via a Michael-type process to the six-membered structure. Olive BPseco substituents control the rate of cyclization from diale to elenolate. This process slows in demethyl oleuropeindiale from demethyl oleuropein through the lack of C11–carboxymethyl. The same process occurs in oleuroside, where the π-bond on C9 shifts to C8. Therefore, complete oleuropein hydrolysis at acetal level of secoiridoid site is rapid when activated by native olive β-glucosidases. Glucoside bond reactions were proposed as conventional paths for olive BPseco hydrolysis [49,50]. This overlooked hydroxyl-aromatic moieties, bis-acetal-nature on Glu–O–C1–O2–C3–, and a third acetal group on glucose residue. Olive BPseco HOMO and HOMO-1 spread over hydroxytyrosil moiety. Removing electrons from oleuropein MOs is energetically easier than adding them. The hydroxyl-aromatic groups could donate electron density under basic and polyphenol oxidase (PPO) catalysis. This Lewis base action forms bonds in free radical and oxidation processes. The high threshold energy of electrophile and nucleophile processes on the hydroxytyrosil site inhibits reactivity, as observed under experimental simulation and actual modes. In that case, hydroxytyrosil moiety results are unreactive on aromatic-OHs of olive BPsecos within the acid-catalyzed environment. The total energy available during simulation experiments exceeds the critical energy for the activation of competing polar processes, i.e., the hydrolytic downstream on glucoside as well as ester groups. These are OBPseco polar reactions, which involve H_3_O^+^ and OH^−^ respectively. They occur earlier on glucoside as well as on ester groups than aromatic–OH of oleuropein reactions due to the high activation process that requires sufficient energy absorption to stretch, bend, and otherwise break the bonds in aromatic rings.

**Figure 7:**
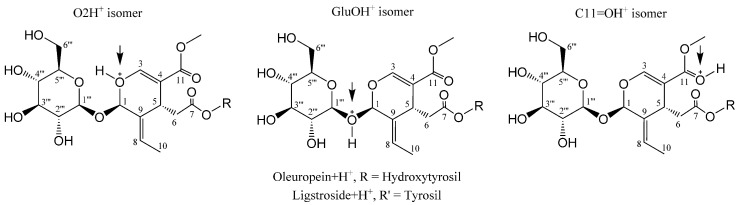
Olive BPseco + H^+^ isomers: oleuropein O2 + H^+^; oleuropein GluO + H^+^; oleuropein C11=O + H^+^. Arrows indicate the position of the positive charges.

**Figure 8:**
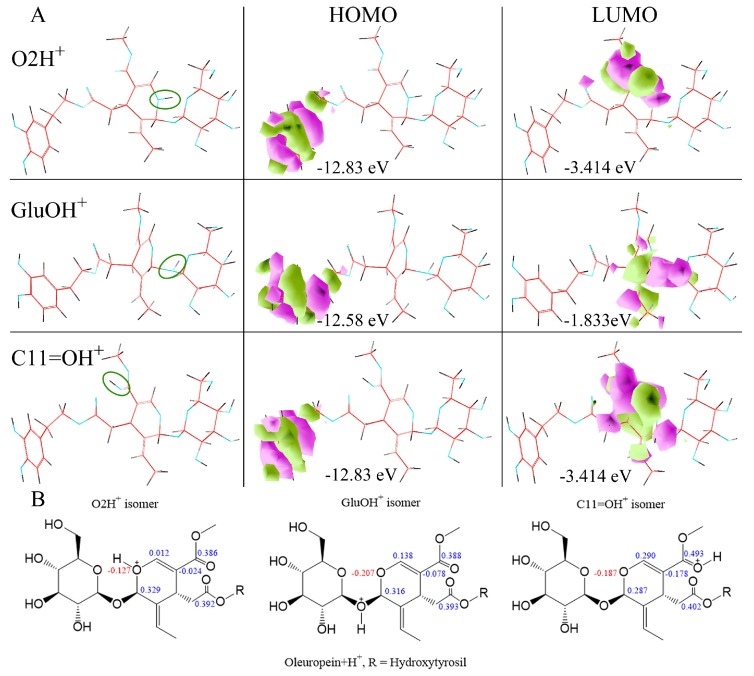
(**A**) Orbital surfaces and energy for HOMO/LUMO of oleuropein + H^+^ isomers, calculated at typed neglected of different overlap (TNDO) level; (**B**) Oleuropein O2 + H^+^; oleuropein GluO + H^+^; oleuropein C11=O + H^+^ excess charges.

Olive BPseco downstream dynamics of polar hydrolysis processes neglect HOMO and HOMO-1 due to the high critical energy required for their reactive interaction with H_3_O^+^ [16]. The first two HOMOs on hydroxyl aromatics play a major role in free radical processes but conflict with other functional group HOMOs under the pre-equilibrium H^+^ of oleuropein simulation and actual paths. They cannot gather sufficient energy to give reaction products after H_3_O^+^ transfers, which leads to competing hydrolytic processes. HOMO-2s relate to polar hydrolysis reactions through interaction with H_3_O^+^ LUMOs, which generate the most probable olive BPseco + H^+^ isomers (Figure 7). In oleuropein downstream hydrolysis, H_3_O^+^ and H_2_O consecutively react to O-basic and C-active sites on the seco-ring, respectively. This requires identification of FMO reagents between oleuropein + H^+^ isomers and H_2_O lone pairs. Looking at olive BPseco + H^+^ isomer FMOs, oleuropein O2 + H^+^, oleuropein GluO + H^+^, and oleuropein C11=O + H^+^ OBPseco MOs, formed during H^+^ transfer pre-equilibrium, provides a tool to predict their reactivity (Figure 8). LUMOs are empty p-orbitals that interact with H_2_O HOMOs. HOMOs/LUMOs of olive BPseco + H^+^ isomers play a prominent role in governing molecular dynamics between reactant molecules. Their energy gaps and ΔEs provide significant reactivity indexes for olive BPseco + H^+^ isomer dynamics, indicating their kinetic stability [52]. Large ΔEs imply greater stability and less reactivity; high stability indicates a low reactivity of olive BPseco + H^+^ isomers, and low ΔE indicates high reactivity.

**Figure 9:**
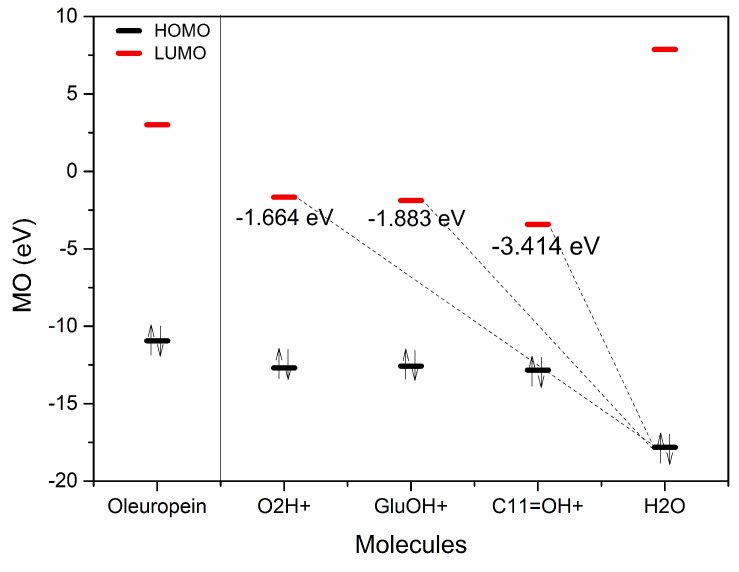
Oleuropein HOMO/LUMO and OLE + H^+^ isomers and H_2_O correlation diagram of their FMO energies.

Likewise, lower energetic HOMO/LUMO gaps indicate the existence of a certain degree of conjugation caused by π-stacking structures –O2–C3=C4–C11=O and –O2–C3=C4–C11=O^+^–H in secoiridoid rings of oleuropein and oleuropein + H^+^ isomers. Additionally, the hydrolytic reactivity of transitional pre-equilibrium cations can be realized by the HOMO/LUMO values of oleuropein + H^+^ isomers (Table 1). ∆E HOMO/LUMO values resulted in oleuropein O2H^+^ isomer > OLEGluO + H^+^ isomer > OLEC11=O + H^+^. The small HOMO/LUMO energy gap (9.416 eV) of the OLEC11=O + H^+^ isomer reveals low kinetic stability and high molecular reactivity. A unimolecular rate determining the transition state of C1–oleuropeinGlu bond cleavage may lead to glucose formation. The alternative glucosyl-carbonium route was generally excluded [53]. The multistep TNDO pathway at reactive moieties proceeds through H_3_O^+^ pre-equilibrium (Figure 1), which protonates oleuropein at aromatic–OH moiety that antagonize the three most probable sites at C1–OGlu, C1–O2–C3, and C4–C11=O Lewis bases to generate olive BPseco isomers (Figure 7). The conventional hydrolysis mechanism invokes H_2_O addition via its weak oxygen base to the secoiridoid ring. As a result, olive BPseco acid-catalyzed and enzymatic breaks may occur via two C–O bonds at the C1 and C3, H_2_O linkage, followed by several H^+^ transfers [16]. This causes the OLEO2H^+^ isomer to be the most reactive, instead of the high molecular reactivity of the OLEC11=O + H^+^ isomer revealed by experimental simulation and computational mapping. Table 1 shows the HOMO/LUMO energy values and their ΔEs of oleuropein and oleuropein + H^+^ isomers. Figure 9 shows the correlation of OLE + H^+^ isomer LUMO with H_2_O HOMO.

ΔE values for energy gaps among OLE + H^+^ isomers and H_2_O reveal that the OLEC11=O + H^+^ isomer provides the dominant interaction with its lowest rate of 1.531 eV and 1.750 eV compared to the OLEGluO + H^+^ and OLEO2H^+^ isomers, respectively. As a result, oleuropein HOMO-2 = −12.13 eV belongs to conjugated system –O2–C3=C4–C11=O<―> –O2^+^=C3–C4=C11–O^−^, which is a good electron donor at the C11=O carbonyl moiety. This explains the Lewis basic character of oleuropein, which destabilized when H^+^ was added due to the bonding disturbance. The conjugated system of the seco-ring and –O2–C3=C4–C11O_2_Me experiences electron withdrawing on the O2 seco-acetal. This effect decreases the exo-OGlu basicity. Partial H^+^ transfer at the transition state and intermediate C1 alkoxy-carbenium stability reduction may explain the low reactivity of olive BPsecos compared to iridoid analogs. Their molecular dynamic decrease relates directly to the hydrolysis rate of analogs lacking –C3=C4– π-bonds [48]. H^+^ transfer reactions prompt toward the basic site onto C11=O carbonyl [16]. The conjugate addition of H_2_O nucleophile on α,β ene-carbonyl moiety of proper olive BPseco + H^+^ isomer follows the thermodynamic product as a 1,4-addition to β-C3, with the largest coefficient at reactive hydrolytic sites C3 = 0.290, C1 = 0.287, and C1”’ = 0.254, while C7 = 0.402 and C11 = 0.493 were unreactive (Figure 8).

Native olive β-glucosidases hydrolyze oleuropein leads to hydroxytyrosil-elenolate via oleuropein aglycone, enol, and dialdehyde. The enzymatic conversion of olive BPsecos can evolve through a sequence of intermediates in an aqueous acidic solution, i.e., intramolecular cyclic hemiacetals, to open aldehyde-enol isomeric forms. These, on the contrary, may be more stable when compared to olive BPseco intramolecular cyclic hemiacetals because of their substitution pattern and planar secoiridoid ring systems. Cyclic hemiacetals stability is highly dependent on the size of the ring, and 6-membered rings are generally favored. Olive BPseco cyclic hemiacetal vs. acyclic forms and consecutive bond rotations and ring closures were controlled by the equilibrium formation among aglycones, the cyclic hemiacetals, and their open isomeric forms. This complex process involves a total of three molecular functions, including C1OH–O2–C3– hemiacetal, C3–enol, and one C1-aldehyde in the first reaction step. The last phase transitions from the enol-aldehyde forms to cyclic elenolates. The first step is the retroreaction to form one biomolecule of cyclic hemiacetal. There is no change in number of biomolecules and no entropy change during the formation of cyclic hemiacetals as they form during intramolecular reactions. Changes in enthalpy in linear molecular cases are generally low and negative because C=O is slightly less stable than 2 × C–O σ–bonds. Change in Gibbs free energy, as ∆G = ∆H − T∆S during acyclic hemiacetal formation, could be positive; however, during the cyclic hemiacetal formation, the change in Gibbs free energy is negative. The same reasoning applies to the conversion of C3–enol and C1–aldehyde isomeric forms into elenolates. The process to olive BPelenolates is a step-by-step mechanism via C5–C9 bond rotation and C3–OH enolate addition to C8=C9 π-bonds. Olive BPseco cyclic hemiacetals undergo reverse equilibria via enol–OH addition to C1–aldehyde carbonyl in isomeric open forms. The molecular dynamics evolve through C3–enol–OH nucleophilic attacks to carbonyl electrophilic C1. C3–enol–OH are weak nucleophiles that react on carbonyl C1 by H_3_O^+^ catalysis on C1=O. Olive BPseco diale and aglycone enol–OH structural isomers involve both C1=O and C3=O carbonyl groups. These undergo intramolecular reactions to form cyclic hemiacetals, which directly release as aglycones by enzyme catalysis.

Notably, the olive milling processes of crushing and malaxation may downgrade total BPs and BPsecos by 50–60%. Approximately 0.6% of the olive bioactives could be transferred to olive oil, while the rest ends up as waste by-products [54]. An increase in water during oil processing resulted in olive BPseco bioactives loss escalation. However, the olive BPseco downstream pathway oleuropein aglycone, oleacein, hydroxytyrosol, and tyrosol were well absorbed in the human gastrointestinal tract via consumption simulation of extra virgin olive oil [55,56,57]. Furthermore, the liver cytochrome P450s, including CYP2D6 and CYP3A4, transformed tyrosol into hydroxytyrosol, which can contribute to therapeutic potential [58]. However, oleuropein was poorly or dose-dependently absorbed, and inhibited CYP3A [59,60,61]. Therefore, the tested olive BPseco downstream pathway in this study emphasizes their stability toward hydrolysis under simulated experimental conditions and TNDO mechanisms that are alike in olive milling processing and in the human gastrointestinal tract. These results may target the location of olive bioactives that can be manipulated to enhance the functional food, nutraceutical, and health potential applications of olives and olive-based products.

## 3. Materials and Methods

Pure oleuropein (89.1%) and isomers, such as oleuroside, were obtained from olive fruits *Olea europaea* L., *Cassanese* cultivar BPseco mixtures as per previous HPLC-MS and NMR protocols [15,51]. pHs and reaction times resembling the human digestion system were simulated under acid and mild basic catalysis using buffering solutions that contained HCl/KCl, pH = 1.5, t = 240 min and T = 37 °C, and KH_2_PO_4_/NaOH, pH = 6.0 and pH = 8.0, with t = 120 min, T= 37 °C. Lower O_2_ tension was simulated by bubbling N_2_ into the solutions for 2 min and then equilibrating again in air. Olive milling process were simulated using CH_3_CO_2_Na/CH_3_CO_2_H at pH = 4.2 for 120 min at 25 °C as well as NaBO_4_/HCl at pH = 8.0 for 240 min at 25 °C [16].

HPLC analysis was performed using a 1100 LC system (Hewlett-Packard, Waldbronn, Germany) with an RP 25 cm × 4.6 mm id, Phenomenex Luna 5 μ C18 column coupled to a UV/VIS detector with a stainless steel loop of 20 µL and a split 1/15 to detector at 280 and 240 nm. Data were processed with both HPChem-Station and online to an ESI/MS interface. Solvents at 0.5 mL/min flow rate were: (A) MeOH, and (B) H_2_O/0.1% CH_3_CO_2_H at pH = 3.3 with selected gradient of 10% (A) and 90% (B), then (A) up to 90% in 60 min [15]. ESI/MS and ESI/MS/MS analyses were performed with a Micromass-Q triple quadrupole, Z-Spray ion source using MassLynx 3.5 software (Micromass, Manchester, UK). N_2_ nebulizing gas was used at 25–30 L/h source and desolvation at 100 °C and 250 °C, respectively, in negative ion mode with capillary 1.9 kV and cone 28 V; for MS/MS/CAD, Ar gas was used with collision energy of 20 eV [15,16].

The semiempirical molecular orbital for the highest occupied molecular orbital (HOMO)/lowest unoccupied molecular orbital (LUMO) evaluation of oleuropein, oleuropein O2 + H^+^, oleuropein GluO + H^+^, and oleuropein C11=O + H^+^ isomers were exploited using the TNDO method, a new semiempirical method that merges molecular mechanics and semiempirical quantum mechanics [62].

## 4. Conclusions

In our study, we found that olive BPsecos were stable under base catalysis and were hydrolyzed under acid and enzyme catalysis. The oleuropein FMO results suggested that downstream pathways competed with free radical and polar molecular dynamics resulting from HO-aromatics and secoiridoid moieties, respectively. Oleuropein HOMO and HOMO-1 could trigger free radical processes, HOMO-2, and LUMO polar reactions of glucoside and ester groups. Oleuropein aglycone opened to diales and rearranged to hydroxytyrosil-elenolate under acid reaction conditions. The downstream pathway olive BPseco HOMOs activated glucoside hydrolysis under native olive β-glucosidases and general acid catalysis. The probed pH, reaction time, and molecular mechanism—proven using TNDO molecular orbital theory—also shed light on how olive BPsecos changed during fruit maturation, how they were hydrolyzed during both olive milling and the intestinal digestion process, and how they were then transferred to olive oil. The results clearly demonstrate that the use of suitable milling process of olives fruits could be exploited towards the complete release of bioactive BPsecos into extra virgin olive oil, which could contribute to improve functional food and nutraceuticals for the wellbeing and health of consumers.

## Figures and Tables

**Table 1:** Highest occupied molecular orbital (HOMO)/lowest unoccupied molecular orbital (LUMO) energy values of OLE and OLE + H^+^ isomers and their ΔEs.

eV	OLE	OLE O2H^+^	OLE GluO + H^+^	OLEC11=O + H^+^	H_2_O
HOMO	−10.94	−12.69	−12.58	−12.83	−17.82
HOMO + 1	−11.75	−14.6	−14.48	−14.76	−19.1
HOMO + 2	−12.13	−15.21	−15.04	−14.98	−20.68
LUMO	3.013	−1.664	−1.883	−3.414	7.871
LUMO-1	3.666	−1.227	−0.4203	0.3386	8.423
LUMO-2	3.76	−0.2421	0.1457	0.6718	–
∆E HOMO/UMO	7.93	11.026	10.697	9.416	–

## Abbreviations

AM1	Austin model 1
BPs	Biophenols
BPsecos	Biophenol secoiridoids
CAD	Collision activated dissociations
FW	Fresh weight
FMO	Frontier molecular orbital
Glu	Glucose
HOMO	highest occupied molecular orbital
HPLC-ESI-MS	High performance liquid chromatography electron spray ionization mass spectrometry
LUMO	Lowest unoccupied molecular orbital
Min	Minute
MS	Mass spectrometry
OLE	Oleuropein
PPO	Polyphenol oxidase
T	Time
TNDO	Typed neglected of different overlap
